# Application of discrete Fourier inter-coefficient difference for assessing genetic sequence similarity

**DOI:** 10.1186/1687-4153-2014-8

**Published:** 2014-05-28

**Authors:** Brian R King, Maurice Aburdene, Alex Thompson, Zach Warres

**Affiliations:** 1 Department of Computer Science, Bucknell University, Lewisburg, PA 17837, USA; 2 Department of Electrical and Computer Engineering, Bucknell University, Lewisburg, PA 17837, USA

**Keywords:** Discrete Fourier transform, Sequence analysis, Sequence similarity

## Abstract

Digital signal processing (DSP) techniques for biological sequence analysis continue to grow in popularity due to the inherent digital nature of these sequences. DSP methods have demonstrated early success for detection of coding regions in a gene. Recently, these methods are being used to establish DNA gene similarity. We present the inter-coefficient difference (ICD) transformation, a novel extension of the discrete Fourier transformation, which can be applied to any DNA sequence. The ICD method is a mathematical, alignment-free DNA comparison method that generates a genetic signature for any DNA sequence that is used to generate relative measures of similarity among DNA sequences. We demonstrate our method on a set of insulin genes obtained from an evolutionarily wide range of species, and on a set of avian influenza viral sequences, which represents a set of highly similar sequences. We compare phylogenetic trees generated using our technique against trees generated using traditional alignment techniques for similarity and demonstrate that the ICD method produces a highly accurate tree without requiring an alignment prior to establishing sequence similarity.

## Introduction

Substantial technological advances continue to be made in modern DNA sequencing instrumentation. Next-generation sequencing (NGS) systems generate genetic and genomic data at unprecedented rates. Methods that can be used to help us understand these data are being researched in earnest. In general, the most common, biologically meaningful approach to understand new sequence data are based on methods that can compare new data against a large set of data that is well understood.

When a new biological sequence with unknown function has been identified, researchers search for the most ‘similar’ sequence in a database of annotated sequence data, under the premise that similar sequences imply similar biological functionality, and in the case of proteins, similar structural characteristics. Similarity between two biological sequences forms the basis for determining whether the sequences are homologous, i.e., there is shared ancestry between them [1]. Phylogenetics, the study of evolutionary relationships between organisms, relies on methods that can quantitatively measure differences between these organisms, with the premise that larger differences between organisms imply a larger span of time before the organisms split from a common ancestor. Phylogenies are most commonly inferred from pairwise comparisons performed on the underlying genetic sequence data obtained from the organisms being analyzed [2,3]. For these and many other reasons, sequence analysis methods are among the most researched and sought after methods in bioinformatics. We encourage the reader to consult a text on biological sequence analysis to learn about existing methods [1,4].

Generally speaking, the predominant methods for biological sequence comparison are based on sequence alignments, such as the popular BLAST and the ClustalW series of methods [5,6]. Alignment methods have represented the *de facto* standard for sequence analysis, comparison, and retrieval. However, the advent of NGS sequencing has pushed traditional alignment methods to their limits. There are numerous user-defined parameters for dealing with gaps and mismatches between sequences, and it is difficult to determine the ideal parameters to achieve an optimal alignment. The computational resources required for these methods can increase quadratically or more with respect to the length of the sequences and the number of sequences being aligned [1]. Moreover, there is an increased risk of errors being introduced with multiple sequence alignments as the average pairwise sequence identity of the data being aligned decreases. Another source of alignment error arises if the order of significant regions in sequences is not conserved [7]. If an optimal alignment has been found, it is difficult to determine an accurate metric of distance between sequences [8]. Despite these challenges, alignment methods continue to be used. With appropriate parameter selection, they excel at visually indicating regions that are highly conserved among many sequences.

To overcome these challenges, there has been increased interest in techniques that can compare sequences without an alignment, referred to as alignment-free methods [7-9]. The most popular alignment-free methods are based on computing various transformations of fixed-length words of length *n* (or *n*-mers, *n*-grams), with common approaches involving computing a frequency vector over all possible *n*-mers for each sequence [2]. Other methods search for a shared set of the longest common subsequences [10]. These methods tend to be among the most efficient, as their computational complexity is linear [9]. However, they may lose valuable information with respect to positioning of important subsequences within the whole sequence. Moreover, like alignment-based methods, they often require multiple runs to select the most ideal parameters.

Digital signal processing (DSP) techniques have been used effectively for efficient searching and comparison of sequential data [11,12]. They are emerging as another alternative alignment-free approach used to analyze both genomic and proteomic data. In order for these data to be processed using DSP techniques, they must be converted to a numeric sequence. There are several numeric representations available, each with their own strengths and weaknesses [13-15]. In the case of DNA, there are a limited number of numeric transformations available. DNA encodes the genetic blueprint of every organism as a sequence over four possible nucleotides, represented as A, C, G, or T. Encoded in DNA are genes, which contain the instructions to make proteins, and intergenic regions, which fill in the large gaps between genes. Within each gene are coding regions (exons) and noncoding regions (introns). The information content, which is critical to understanding the biological function of the gene, is hidden in the coding regions in the gene. Coding regions are comprised of codons, nucleotide triplets that code for individual amino acids, and represent a very small portion of the entire genome. In the human genome, only about 5% of it contains coding instructions. These complexities make the process of identifying genes and coding regions within these genes a daunting task.

Proteins have more choices of possible numeric transformations available, owed in part to the physicochemical properties of amino acids. Proteins themselves are long polypeptide chains of amino acids. There are 20 possible amino acids that exist in proteins, each having many physicochemical properties, such as hydropathy, charge, and solubility. These properties provide useful numeric representations for protein sequences, making a translation to a numeric sequence a relatively easy process. For example, the MAFFT method is a protein sequence alignment method that converts converted proteins into numeric sequences that represent the polarity and volume values of each amino acid residue in the proteins being aligned [16].

Regardless of the numeric transformation chosen, preservation of information content in the sequence is critical. This is perhaps one reason for the most common representation of a DNA, the binary indicator sequence, also commonly known as the Voss representation [17]. In this representation, each DNA sequence is transformed into a sequence of binary occurrence vectors. (This is the transformation used in our research, and is described in detail in the Methods and materials section). Some methods use variations of the binary indicator sequence. For example, Afrexio et al. introduced a variant of the Voss representation that converts the occurrence vector into a vector of inter-nucleotide distances [18]. There is a wide range of transformations available [13]. Hota et al. analyzed the performance of several common DNA to numerical mapping techniques. They provide a good description of each transformation method used in practice [19].

DSP based methods have continued to emerge in recent years for the purpose of genomic analysis. The most prevalent use has been to locate reading frames in DNA, as well as different regions in the genome, including genes and coding (or exon) regions within these genes [14,20,21]. Sharma et al*.* analyzed the performance of several DNA mapping schemes for detecting the coding region of genes [15]. DSP techniques have been used to address other problems in genomics and proteomics. For example, methods have been developed for splice site detection within the gene [20], the identification of active sites in a protein using Morlet wavelets [22] and identification of acceptor splicing sites and the visual identification of patterns and motifs in DNA through spectral analysis [14,23].

Regardless of the domain, the field of digital signal processing has provided a plethora of methods for analyzing sequential data. Most methods use variations of the Fourier transform [24], with the discrete Fourier transform (DFT) being among the most popular signal processing technique [25,26]. Typically, the fast Fourier transform (FFT) is used to compute the DFT, as it is among the most computationally efficient algorithms for this purpose [24]. These transforms have been successfully used for general sequential data comparison and retrieval [11,12], and are readily suitable for biological sequence comparison, owed to the inherent discrete, symbolic nature of biological sequences [14,27,28]. In fact, FFTs have been used to analyze DNA data before [20,29,30]. In addition to some of the methods listed previously, Cheever et al. measured the cross correlation of two DNA sequences to explore significant regions of similarity between the DNA, where the cross correlation was computed using a FFT [31]. The FFT has also been used for protein sequence alignments in the MAFFT method [16].

There have been many DSP-based methods introduced in recent years for biological data analysis; however, very few were designed to report a biologically relevant measure of evolutionary distance between sequences being analyzed, particularly when a large number of sequences are being analyzed. Multiple sequence alignments have been used successfully for this purpose, but these methods can be computationally expensive and are prone to errors, particularly as the set of sequences being analyzed increase in size and diversity. We developed a novel signal processing technique that characterizes genetic sequence data through a simple transformation of the coefficients generated by the DFT of a specific numeric representation of the original DNA sequence. In our work, we compute a transformation on the set of coefficients generated that we call the *inter-coefficient difference* or ICD. We show that this characterization effectively produces a signature for a given sequence and can be used to compare genetic sequences among different species. The ICD method provides comparisons between genes from evolutionarily distant species, as well as subtle variants from identical genes from the same species. We demonstrate its effectiveness through analysis of datasets that have different levels of pairwise similarity. The method effectively generates a pairwise distance matrix representing the level of similarity between each genetic sequence with remarkable running times. The resulting matrix can be used to induce a dendrogram representing phylogenetic relationships between species from which the sequences were obtained. Our results show that we produce alignment-free dendrograms that are highly similar to those trees produced using alignment-based techniques and other alignment-free methods.

## Methods and materials

Our method is based on the application of the DFT to four numeric sequences that are derived from the original DNA sequence. We use a binary indicator sequence representation of a DNA sequence, which is among the most popular numeric representation used in this area in literature [17,20]; it allows for an easy transformation from the original sequence on which many DSP and other numeric transformations can be computed [18,20,27].

### The inter-coefficient difference

Let *S* represent a set of DNA sequences, where *s*_*i*_ represents an arbitrary sequence in *S*. Each DNA sequence *s*_*i*_ is defined over the alphabet. Let *N* be the length of the longest sequence in *S*. Each sequence *s*_*i*_ in *S* goes through a series of transformations to produce the corresponding ICD vector. The first transformation computes a unique binary indicator sequence from *s*_*i*_. Next, we apply the DFT on the indicator sequence, yielding a vector of coefficients. Basic mathematical transformations are applied to the coefficient vector, resulting in the ICD vector. The details of this algorithm are given below.

For a given sequence *s*_*i*_, we define four binary indicator sequences *x*_*A*_[*n*], *x*_*C*_[*n*], *x*_*G*_[*n*], and *x*_*T*_[*n*], which indicate the presence (i.e., a 1) or absence (i.e., a 0) of a symbol in *s*_*i*_ at position *n*. Each indicator sequence is padded with zeros to ensure that every indicator sequence in *S* has an identical length of *N*. Zero padding is a common technique with FFT computations that can increase the spectral resolution and can increase the efficiency of the computation when the length of the original sequence is padded to a power of 2 [26]. For example, let *s*_*i*_ = GACGACTCAT, which has a length of 10. However, suppose that *N*, which is the length of the longest sequence in S, is 12. Then:

$$s_{i}=\text{GACGACTCAT}$$

$$x_{A}=010010001000$$

$$x_{C}=001001010000$$

$$x_{G}=100100000000$$

$$x_{T}=000000100100$$

For each indicator sequence, we compute the DFT, which converts the finite-length sequence *x*_*A*_[*n*] into a series of coefficients *X*_*A*_[*k*] resulting from the DFT computation, defined in Equation 1:

(1) $$X_{A}\left[k\right]=\sum_{n=0}^{N-1}x_{A}\left[n\right]e^{-j\left(\frac{2\text{πnk}}{N}\right)}\;k=0,1,\ldots ,N-1$$

The coefficients produced are complex, and thus the absolute value of each coefficient is computed, yielding a series of real valued numbers. *X*_*A*_[0] represents the number of 1 s in the indicator sequence *x*_*A*_. It is discarded because it is substantially larger than all other coefficients and is highly dependent on the length of the original unpadded sequence. We retain coefficients XA1,XA2,…,XAN2, eliminating half of the coefficients because of the symmetric nature of the coefficients produced by the DFT [26]. The remaining coefficients are denoted as vector $X_{A}^{*}$. We normalize $X_{A}^{*}$ by dividing by its Euclidean norm, $\left\|X_{A}^{*}\right\|$, resulting in **X**_*A*_. Equation 2 illustrates this transformation, introducing variable η for simplicity:

(2) η=N2XA*=XA1,XA2,…,XAηXA=XA*XA*

For each vector **X**_A_, we compute the inter-coefficient difference of **X**_A_, denoted ICD(**X**_A_), by computing the difference between each adjacent number in the sequence as shown in Equation 3:

(3) $$\mathrm{ICD}\left(X_{A}\right)=\left[X_{A}\left[2\right]-X_{A}\left[1\right],X_{A}\left[3\right]-X_{A}\left[2\right],\ldots ,X_{A}\left[\eta \right]-X_{A}\left[\eta -1\right]\right]$$

The same computations are repeated for indicator sequences *x*_*C*_, *x*_*G*_, and *x*_*T*_, yielding vectors **X**_*C*_, **X**_*G*_, and **X**_*T*_ separately.

For example, continuing from our previous example indicator sequence, *x*_*A*_ = 010010001000, and *N* = 12. We apply Equations 1 and 2 above on *x*_*A*_, which computes the DFT on *x*_*A*_ and normalizes it, resulting in the vector of coefficients **X**_*A*_:

$$X_{A}^{*}=\left[0.5176,1.0000,2.2361,1.7321,1.9319,1.0000\right]$$

$$\left\|X_{A}^{*}\right\|=3.7417$$

$$X_{A}=\left[0.1383,0.2673,0.5976,0.4629,0.5163,0.2673\right]$$

Then, the inter-coefficient difference of **X**_A_ is computed, resulting in:

$$\text{ICD}\left(X_{A}\right)=\left[0.1289,0.3304,-0.1347,0.0534,-0.2490\right]$$

The ICD of each coefficient vector resulting from vectors **X**_*C*_, **X**_*G*_, and **X**_*T*_ is concatenated to produce a single numeric vector, denoted **X**.

$$X=\left[\text{ICD}\left(X_{A}\right)\text{ICD}\left(X_{C}\right)\text{ICD}\left(X_{G}\right)\text{ICD}\left(X_{T}\right)\right]$$

It is important to mention that all ICD vectors will have an equal length for every sequence in *S*, regardless of the length of the original sequence. Each indicator sequence transformation is padded to have a length of *N*, which is the length of the longest sequence in *S.* The final concatenated vector **X** will have a length of 4N2=4η.

### Establishing distance between DNA sequences

Given two arbitrary DNA sequences, *s*_1_ and *s*_2_ in set *S*, we can compute the ICD transformation yielding numeric vectors **X**_1_ and **X**_2_, respectively. A single numeric value that represents a measure of biological distance is computed from these vectors by computing the correlation between the two vectors. We compute Dist (**X**_1_, **X**_2_), a single measure of distance between the ICD vectors, as follows:

(4) $$\text{Dist}\left(X_{1},X_{2}\right)=1.0-\frac{\sum_{i=1}^{4\eta }\left(X_{1}\left[i\right]-{\bar{X}}_{1}\right)\left(X_{2}\left[i\right]-{\bar{X}}_{2}\right)}{\sqrt{\sum_{i=1}^{4\eta }{\left(X_{1}\left[i\right]-{\bar{X}}_{1}\right)}^{2}\sum_{i=1}^{4\eta }{\left(X_{2}\left[i\right]-{\bar{X}}_{2}\right)}^{2}}}$$

Equation 4 is 1.0 minus a standard correlation calculation between two sets of data. We know that a standard correlation falls in the range [−1.0, 1.0], where −1.0 is a perfect negative correlation and 1.0 is a positive correlation. Two vectors of identical values would have perfect positive correlation, and thus their Dist calculation would be 0.0, implying that there is no distance between them. A value of 2.0 is perfect negative correlation, implying opposing numerical trends around the means.

### Data

To test the efficacy of this method, we assembled two sets of DNA data. Our first set consisted of mRNA insulin sequences from 19 different animals, called *INS19* (Table 1). Insulin is an important hormone found throughout the animal kingdom for regulating carbohydrate and fat metabolism and for managing glucose levels in the blood. All sequences were downloaded from NCBI's RefSeq database (http://www.ncbi.nlm.nih.gov/refseq/). This dataset was chosen to measure the ability of the method to assess pairwise similarity over a set of sequences that have highly conserved regions in its genetic sequence owed to its similar function among all species while exhibiting substantial regions of low conservation in proportion to the evolutionary distance between species. The length of the sequences in the data ranged between 291 and 774 nucleotides in length.

**Table 1:** mRNA insulin sequences from 19 animal species in the INS19 dataset

**Species**	**Common name**	**Accession**	**Length**
*H. sapiens*	Human	NM_000207	469
*P. troglodytes*	Chimp	NM_001008996	416
*O. baboon*	Olive baboon	XM_003909376	505
*M. fascicularis*	Monkey	J00336	392
*B. taurus*	Cow	NM_173926	434
*S. scrofa*	Pig	NM_001109772	435
*G. gallus*	Chicken	NM_205222	453
*C. familiaris*	Dog	NM_001130093	463
*F. catus*	Cat	AB043535	420
*C. procellus*	Guinea pig	NM_001172891	442
*C. cristata*	Star-nosed mole	XM_004695041	291
*E. telfairi*	Hedgehog	XM_004717178	327
*M. auratus*	Hamster	XM_005064148	450
*O. cuniculus*	Rabbit	NM_001082335	433
*D. rerio*	Zebrafish	AF036326	468
*P. buchholzi*	Butterfly fish	AF199588	459
*C. chitala*	Clown knifefish	AF199586	375
*F. albicollis*	Flycatcher	XM_005046804	324
*X. laevis*	Clawed frog	NM_001085882	774

Our second set of data was chosen to test the ability of the method to accurately distinguish subtle differences among a large set of sequences from the same gene obtained from the same viral species. To this end, we selected 60 influenza type A sequences collected from the NCBI Influenza Virus Sequence Database (http://www.ncbi.nlm.nih.gov/genomes/FLU/). Influenza is an RNA virus that affects a wide range of mammals and birds; in extreme cases, it can lead to death. Influenza A viruses are broken down into different subtypes that are named based on two specific proteins that are on the surface of the virus: hemagglutinin (HA) and neuraminidase (NA). There are 17 types of the HA protein and 10 types of neuraminidase NA protein. Each virus receives a designation labeled H*x*N*y*, where *x* represents a specific subtype of the HA gene and *y* represents a subtype of the NA gene in the virus. Our dataset, denoted FLU60, contains 60 examples of avian influenza sequences (influenza sequences known to affect birds) for the HA gene only, collected from various locations in the United States between January and July of 2010. Avian flu strands were selected because all known subtypes of influenza A can affect birds. The length of all sequences in FLU60 ranged between 1,683 and 1,746 nucleotides in length. The frequency of influenza A subtypes in the dataset are detailed in Table 2. The most dominant variant in the data is H4N6 at 25 examples, with H3N*x* variants coming in second. Because we collected only examples of the HA gene, only the H*x* part of the subtype name should play a role in determining similarity. Additional file 1: Table S1 has detailed information about the dataset, including the accession number, subtype, date and place that specimen was acquired, and the length of each sequence [see Additional file 1].

**Table 2:** Avian influenza A subtype frequency in FLU60

**Influenza A subtype**	**Frequency**
**H1N1**	3
**H1N3**	1
**H3N1**	1
**H3N6**	1
**H3N8**	13
**H4N6**	25
**H6N1**	2
**H7N3**	6
**H9N2**	1
**H10N7**	4
**H11N9**	2
**H12N5**	1

## Results

To assess the capability of the ICD method to measure sequence similarity, we generated a dendrogram based on a hierarchical clustering using the unweighted pair group method average (UPGMA) method for constructing the tree. This was performed for both *INS19* and FLU60 datasets. For comparative purposes, we computed an all-against-all pairwise global alignment using the standard Needleman-Wunsch algorithm for each set of sequences being tested [32], utilizing a uniform nucleotide substitution matrix (as defined by the nuc44 function in the Matlab® Bioinformatics Toolbox) for the purpose of finding the best alignment. Though computing a pairwise alignment for all possible pairs of sequences is computationally expensive, this will yield a superior alignment than any single multiple sequence alignment (MSA), as it significantly reduces the likelihood of introducing alignment errors that result from an MSA. The distance between each pair of aligned sequences was computed by measuring the proportion of sites in the alignment at which the two sequences are different, yielding a score of 1 for entirely dissimilar sequences and 0 if they were identical. This distance measure yields identical groupings to those that are generated directly from the alignment score itself but has a comparative advantage of producing numbers that are in an identical range to the distance values that are produced with the ICD method. ICD uses a correlation coefficient between coefficient differences and likewise always produces a distance value between 0 and 1. We also compared our results to an alignment-free sequence comparison method called feature frequency profile (FFP), which is a popular tool for phylogenetic analysis [2]. We used default parameters on all FFP tools to generate a tree, with the exception of word size; we evaluated word sizes between 6 and 20 and determined that a word size of 16 achieved results that produced the most biologically correct phylogenetic groupings. Finally, the Robinson-Foulds (RFdist) tree distance metric is computed on the INS19 test using the treedist function in the phangorn package in R [33,34]. RFdist is computed between all combinations of pairs of trees to assist in measuring tree similarity.

### ICD method on INS19 dataset

**Figure 1:**
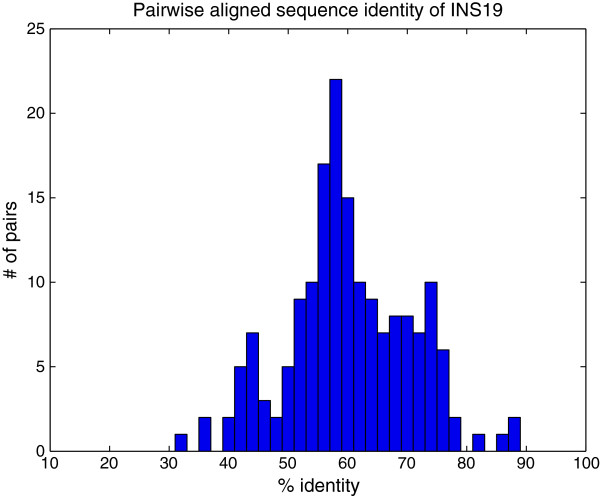
**Histogram of observed sequence identity over all pairs of aligned sequences in INS19 dataset.** The percent identity is computed for all possible pairs of sequences in the INS19 dataset. Most data averaged between 55% and 75% sequence identity.

**Figure 2:**
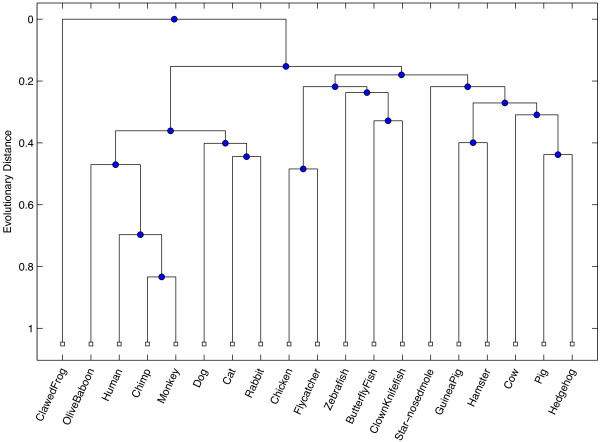
**ICD-based dendrogram for INS19.** This figure shows the resulting dendrogram generated based on the ICD method applied on the ICD19 dataset, which contains mRNA sequences taken from 19 different eukaryotic species for the insulin (INS) gene.

**Figure 3:**
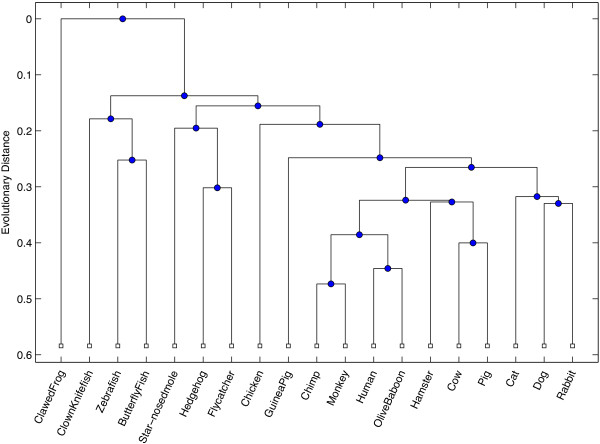
**Alignment-based dendrogram for INS19*.*** This figure shows the resulting dendrogram generated from phylogenetic relationships inferred from pairwise alignments computed over all pairs from the INS19 dataset, which contains mRNA sequences taken from 19 different eukaryotic species for the insulin (INS) gene.

**Figure 4:**
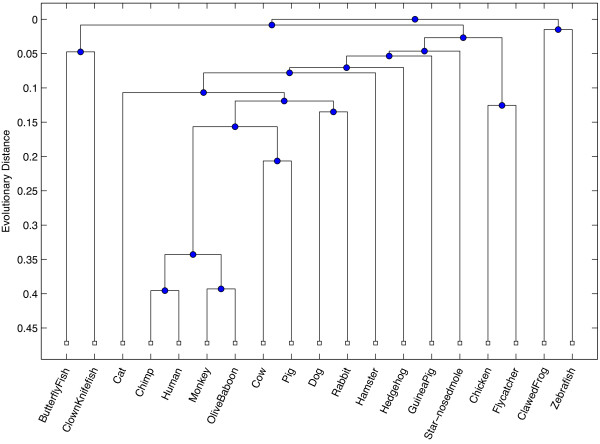
**Alignment-free-based dendrogram using FFP **[2]** method for INS19.** This figure shows the resulting dendrogram generated from phylogenetic relationships inferred using the FFP method on the INS19 dataset, which contains mRNA sequences taken from 19 different eukaryotic species for the insulin (INS) gene.

Our first test was conducted to measure the ability for the ICD method to accurately assess similarity between sequences that are relatively divergent, where the data was collected from a wide range of eukaryotic species. The INS19 dataset contains data from the insulin gene, taken from 19 species in the eukaryotic kingdom. The range of pairwise sequence identity after alignment ranged between 32% and 89% identity, with an average observed percent identity at 60% (see Figure 1). A dendrogram was built based on the pairwise similarity computed from the ICD method and is shown in Figure 2. For comparison purposes, an all-against-all pairwise global alignment (denoted AAP) was performed on all sequences, and a dendrogram was built revealing the relationships between the sequences based on the alignment. A dendrogram was also computed based on the alignment-free FFP method [2]. The resulting dendrograms from each of these comparative methods are shown in Figures 3 and 4, respectively.

All trees exhibit strong similarities within major groupings, closely resembling phylogenetic relationships observed in nature, with some subtle, yet biologically significant differences between each method. In particular, both ICD and AAP methods place monkey and chimp as the most similar among all species, whereas FFP places human and chimp as most similar. All methods suggest the African clawed frog as most distant from others species used in this study. The FFP method grouped the zebrafish with the clawed frog, whereas the ICD and AAP methods correctly cluster all three fish species. The AAP method grouped a hedgehog, a type of rodent, with a flycatcher, a type of bird. In contrast, the ICD and FFP methods correctly grouped the flycatcher with a chicken, which are both types of birds, and the hedgehog with other similar mammals. The AAP method grouped the hamster, a rodent, with the cow and pig, which are both even-toed ungulates; the FFP method fared a bit better, placing a hamster between a rabbit and hedgehog. In contrast, the ICD method correctly grouped the hamster with the guinea pig, which are both rodents.

The RFdist distance metric was computed between all pairs of trees. The RFdist between the ICD and FFP phylogenetic trees is 26, between ICD and AAP is 24, and between FFP and AAP is 22. These values suggest that, though the trees have similar groups, they have a relatively equal number of different partitions of data that are implied by each tree, with the final tree produced by the ICD method being only slightly more similar to the tree produced by the all-against-all pairwise alignment than the FFP method.

### ICD method on FLU60 dataset

**Figure 5:**
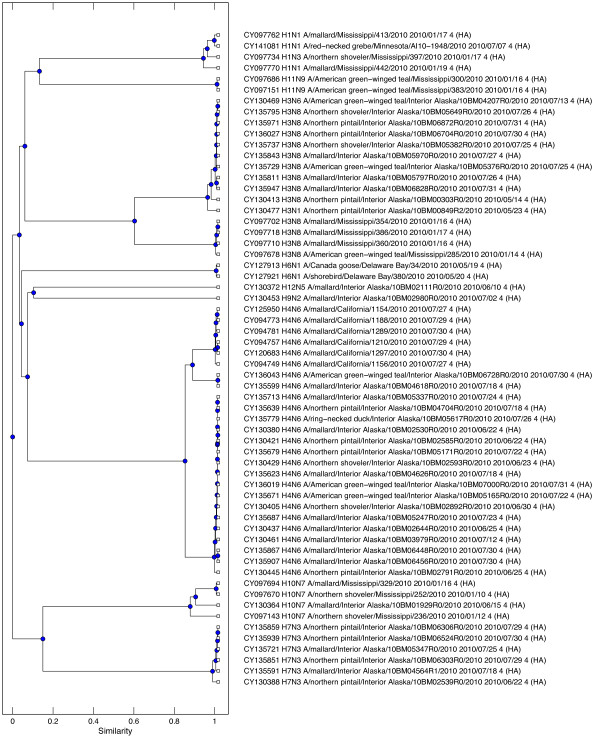
**ICD-based dendrogram for FLU60.** This figure shows the resulting dendrogram generated based on the ICD method applied on the FLU60 dataset, which contains 60 sequences of the HA gene of different subtypes of avian influenza type A.

Our next test was conducted on the FLU60 dataset, which contains 60 DNA sequences of the HA gene from avian influenza A virus. Conducting an all-against-all pairwise alignment revealed a pairwise sequence identity range of 57% to 99.9%, with an average identity of 70.5%. Additional file 1: Figure S1 shows a histogram revealing the sequence identity over all pairs of sequences (see Additional file 1). We performed identical analyses on these data to the analyses performed with the INS19 data, resulting in dendrograms from each method. The dendrogram for the ICD method is shown in Figure 5. The dendrograms for the AAP and FFP methods are shown in Additional file 1: Figures S2 and S3 (see Additional file 1). The RFdist metric was not measured for this test.

Close evaluation of these dendrograms will reveal remarkably similar groupings among each individual subtype of influenza A. We were pleased to see that all influenza HA subtypes were grouped together correctly by all methods. In particular, in the case of H3 and H4 subtypes, all three methods indicated two very distinct strains. H3 is divided into a strain that hit Mississippi and one that hit Alaska. H4 was divided into three distinct strains, with all methods agreeing on the divisions. When looking at similarity between subtypes, all methods group together influenza A subtype H7 with H10, suggesting that each of these groups share a common ancestor. However, they differ slightly on the ancestry relationships between H9, H11, and H12. These findings, as well as most of the other relationships observed in this study, are confirmed by Air's work on sequence relationships in the hemagluttinin genes of 12 different variants of influenza A [35]. The methods differ on the divergence point of subtype H6; the AAP and FFP methods suggest that H6 and H1 have a common ancestor, whereas the ICD method suggests that H6 diverged much earlier from a subgroup consisting of H4, H9, and H12. The AAP and FFP method are closer to the similarity observed in Air's work. However, the level of similarity computed by the ICD method between H6 and subgroup H4, H9, and H12 is remarkably similar to the alternative group H1, H11, and H3, suggesting that the common ancestor could have been from either group.

The execution times were recorded for each of the methods we investigated. In addition, we included the timing results of ClustalW2 [36] and Clustal Omega [37], which are two other popular multiple sequence alignment methods widely used today. All methods were run on a laptop computer running Mac OS X 10.9 with a 2.2 GHz Intel Core i7 processor equipped with 16 GB of memory. The ICD and AAP methods were run as Matlab® applications, while FFP, ClustalW2, and Clustal Omega were compiled and installed as native applications. All methods were executed multiple times with similar loads. The first time was discarded to eliminate bias resulting from file system latency. All methods ran under one second of execution time on the INS19 dataset. This is not surprising given the small size of the dataset and the short length of each sequence. The FLU60 dataset provided a much more informative comparison. Table 3 shows the results for all five methods tested. The results clearly indicate the strength of alignment-free methods with respect to running times. Among the alignment-free methods, the ICD method outperformed FFP, despite the fact that it is running within the Matlab® framework. This suggests that even better execution times may be observed with the ICD method if it was redesigned as a native application.

**Table 3:** Observed execution time for FLU60

**Method**	**Exec time (sec)**
**Clustal W**	157.0
**Clustal Omega**	27.0
**Pairwise alignment**	53.8
**FFP**	7.1
**ICD**	0.2

## Discussion

The binary indicator representation of DNA is a common representation to use on methods that treat DNA as a digital signal [19]. Some suggest that this representation is common because it inherently retains the important three-base periodicity which is important for detecting coding regions in DNA [15]. Some methods make interesting transformations to the original indicator sequence, such as the inter-nucleotide distance utilized by Afreixo et al. [18], with the goal of strengthening signals in the original digital signal that are discriminatory between different sequences. We applied the DFT on the indicator sequence. The DFT is a common DSP technique on digital signals and has been used in other methods for DNA sequence analysis. Each coefficient of the DFT represents a cross correlation of the entire input sequence and a complex sinusoid at a specific frequency, notably *k*/*N*[25]. As noted, DFTs have been successfully used in detecting coding regions of genes, where a strong peak is observed at frequency *N*/3. Our work was largely motivated by an interest in investigating how the differences of the magnitudes of the sinusoids between adjacent frequencies might improve sequence characterization in DNA. The ICD method presents a novel use of the DFT by computing the inter-coefficient difference from the resulting set of coefficients computed by the DFT transformation. The analysis presented here demonstrates its potential as a viable alternative approach toward DNA sequence analysis. In particular, the ability to distinguish differences between sequences having both low and high measures of homology, without computing an alignment, is particularly useful, compared to the challenges from computing multiple sequence alignments over large amounts of biological sequence data.

One may wonder about the likelihood of two different sequences producing an identical ICD vector. The critical part of the ICD method is the DFT. Two different DNA sequences will produce a unique set of coefficients, and likewise, our ICD transformation applied to these coefficients is thus also unique, except under one condition: when one sequence is a rotational shift of the other, and both of these sequences represent the longest sequences in the set of DNA being analyzed, meaning, there will be no zeros appended to either sequence. For example, if the sequences GACGACTCAT and TGACGACTCA (the second sequence is equivalent to the first sequence right-shifted by 1 with rotation) were both in the set of DNA being analyzed and were the longest sequences in the data, they will both yield the same $X_{A}^{*},X_{C}^{*},X_{G}^{*}$, and $X_{T}^{*}$ vectors. However, the likelihood of two biologically meaningful DNA sequences being an entire rotational shift of the other is highly unlikely, particularly when analyzing entire genes. If this event were to actually occur in nature, then our method will yield these sequences as being identical, rightfully drawing the attention of the researcher.

Though we tested several datasets to determine the efficacy of the method, it does have a limitation worth noting. The ICD method works well when assessing similarity of identical genes over many different species, such as the INS19 dataset. It also works well when assessing similarity over many variants of the same gene from the same species, such as the FLU60 dataset. However, for evaluating the similarity over large, genomic regions or entire genomes from different species, the ICD method is limited. The reason for this is due to the requirement of padding zeros to the indicator sequences to ensure all sequences have equal length. Sequences that are significantly shorter than the longest sequence will likewise have a substantial vector of zeros padded and thus will yield comparatively poor ICD vectors.

The results of the ICD method compared favorably to other methods tested. In fact, we observed examples in the INS19 test where the ICD analysis yielded a more phylogenetically correct tree than those produced from other methods tests, backed up by simple phylogenetic relationships observed in any biological text. We opted to perform an all-against-all pairwise alignment over a multiple sequence alignment to ensure the highest degree of accuracy of the measure of similarity of alignments. In the FLU60 data, the ICD methods ability to detect the correct measure of similarity among even those sequences that had a high measure of pairwise sequence identity was remarkable.

A significant disadvantage of alignment-based sequence comparison methods is that they assume that important regions in the genetic sequence will follow the same order between similar species. However, as noted by Pinello et al., this is not always the case [7]. As a method based on the DFT, the ICD method capitalizes on recurrent patterns, regardless of the position of those patterns in the whole sequence. It is robust to possible reordering of conserved regions between genetic sequences.

The ICD method offers a significant advantage over alignment and alignment-free methods by eliminating the need for parameters. Other methods often require multiple runs to determine the best parameter set. In comparison, our ICD method is a pure mathematical, alignment-free transformation that requires no user-defined parameters prior to the analysis.

Depending on the alignment algorithm chosen, the running time to compare *m* sequences of length *n* and produce a tree based on alignment methods can vary between *O*(*m*^2^*n*^2^) for ClustalW [6] to as high as *O*(*n*^*m*^) for dynamic programming approaches. More recently, Clustal Omega implemented substantial improvements over its predecessors in the Clustal family, improving the running time to O(*nm* log *m*), making it suitable for large-scale multiple sequence alignments. Alignment-free methods often have a performance advantage, particularly those that are based on *k*-mer frequencies. These methods can be run in *O*(*knm*) time, noting that selection of word size will have an effect on the final performance. This is particularly important for DNA, which requires longer word lengths for meaningful results. In contrast, the FFT runs in O(*mn* log *n*), suggesting that it is an efficient technique, comparable with other alignment-free methods. Our results in Table 3 confirm the theoretical running times, with the alignment-free methods having a superior advantage over the alignment methods.

Alignment-based methods have their advantages. In particular, an alignment will often produce a better *absolute* value of evolutionary distance between sequences by incorporating a substitution matrix such as BLOSUM62. In contrast, it is relatively difficult to infer a precise measure of evolutionary distance from alignment-free methods, and this is particularly true of the correlation computed from the ICD vector. This is not uncommon, as this is a limitation with any phylogenetic approach that involves computing a distance matrix based on sequence homology. Despite this limitation, most of the relative distances observed between species in the INS19 dataset and between different variants of avian flu in the FLU60 dataset were consistent with the alignments produced. More interestingly, we demonstrated a few differences between the results from the methods applied to the INS19 data, where the ICD approach produced evolutionary relationships that were more consistent with our biological understanding of evolution among species that the other approaches we evaluated failed to capture.

The use of a correlation coefficient for distance is part of the novel approach in this work. Even though the theoretical value of the Dist computation is [0.0, 2.0], all pairs of sequences analyzed had values between 0.0 and 1.0. In other words, sequences were either found to have a strong positive correlation, which is implied for Dist values near 0, or no correlation, for Dist values near 1.0. Our observations on all tests never observed Dist computations of more than 1.0. If this had happened, it would have implied that the two biological sequences being tested had a negative correlation with respect to their ICD vector. From a biological viewpoint, different species, genes, or even different variants within the same genes arise due to evolution; more specifically, due to selective pressures placed on the genome to become more ‘fit’ than its ancestors. The processes behind natural selection that are so important for breeding new species and genetic functions are not random. However, the underlying genetic mutations that occur over eons are generally considered to be random events [38]. The fact that we never observed a negative correlation might offer a metric to numerically confirm the random nature of evolution. This needs further investigation over a much larger set of genetic data to draw any conclusions.

## Conclusions

In this paper, we present a novel use of the discrete Fourier transform to establish sequence similarity through incorporating a simple transform of the coefficient vector. We demonstrated its efficacy on two datasets designed to measure the method's capability on establishing similarity among datasets with different levels of sequence identity. The ICD approach produced a high quality dendrogram representing phylogenetic relationships of sequences with different levels of sequence identity. Our results were nearly identical with those obtained using traditional alignment-based approaches.

## Abbreviations

AAP: all-against-all pairwise global alignment; DFT: discrete Fourier transform; DNA: deoxyribonucleic acid; DSP: digital signal processing; FFP: feature frequency profile method; FFT: fast Fourier transform; FLU60: dataset of 60 variants of avian influenza; HA: hemagglutinin (an influenza gene); ICD: inter-coefficient difference; INS19: dataset of the insulin gene from 19 species; MSA: multiple sequence alignment; NA: neuraminidase (an influenza gene); RFdist: Robinson-Foulds tree distance metric; UPGMA: unweighted pair group method average; ⌊*x*⌋: the floor of *x*; ‖**X**‖: the euclidean norm of vector X.

## Competing interests

The authors declare that they have no competing interests.

## Supplementary Material

Additional file 1 **Application of the discrete Fourier transform on DNA for sequence similarity. Table S1.** Avian Flu Sequences (FLU60). **Figure S1.** Histogram of % identity in FLU60. **Figure S2.** Alignment based dendogram for *FLU60*. **Figure S3.** FFP based dendogram for *FLU60*. Click here for file

## References

[B1] Durbin R Eddy SR Krogh A Mitchison G Biological Sequence Analysis: Probabilistic Models of Proteins and Nucleic Acids 1998 Cambridge University Press, Cambridge, UK 356

[B2] Sims GE Jun S-R Wu GA Kim S-H Whole-genome phylogeny of mammals: evolutionary information in genic and nongenic regions Proc Natl Acad Sci U S A 2009 106 17077 82 10.1073/pnas.0909377106 19805074 PMC2761373

[B3] Phillips A Janies D Wheeler W Multiple sequence alignment in phylogenetic analysis Mol Phylogenet Evol 2000 16 317 30 10.1006/mpev.2000.0785 10991785

[B4] Samuelsson T Genomics and bioinformatics: an introduction to programming tools for life scientists 2012 1 Cambridge University Press, Cambridge, UK 356

[B5] Altschul S Gish W Miller W Myers E Lipman D Basic local alignment search tool J Mol Biol 1990 215 403 410 10.1016/S0022-2836(05)80360-2 2231712

[B6] Thompson JD Higgins DG Gibson TJ CLUSTAL W: improving the sensitivity of progressive multiple sequence alignment through sequence weighting, position-specific gap penalties and weight matrix choice Nucleic Acids Res 1994 22 4673 4680 10.1093/nar/22.22.4673 7984417 PMC308517

[B7] Pinello L Lo Bosco G Yuan G-C Applications of alignment-free methods in epigenomics Brief Bioinform 2013 15 419 430 24197932 10.1093/bib/bbt078 PMC4017331

[B8] Vinga S Almeida J Alignment-free sequence comparison–a review Bioinformatics 2003 19 513 523 10.1093/bioinformatics/btg005 12611807

[B9] Bonham-Carter O Steele J Bastola D Alignment-free genetic sequence comparisons: a review of recent approaches by word analysis Brief Bioinformonline only, published July 31, 2013 10.1093/bib/bbt052 PMC4296134 23904502

[B10] Domazet-Lošo M Haubold B Alignment-free detection of local similarity among viral and bacterial genomes Bioinformatics 2011 27 1466 72 10.1093/bioinformatics/btr176 21471011

[B11] Rafiei D Mendelzon A Efficient Retrieval of Similar Time Sequences Using DFT Proceedings of 5th International Conference of Foundations of Data Organization – FODO '98 1998 Kobe, Japan 249 257

[B12] Wu Y-L Agrawal D El Abbadi A A comparison of DFT and DWT based similarity search in time-series databases Proc. ninth Int. Conf. Inf. Knowl. Manag. - CIKM ’00 2000 ACM Press, New York, USA 488 495

[B13] Cristea PD Conversion of nucleotides sequences into genomic signals J Cell Mol Med 2002 6 279 303 10.1111/j.1582-4934.2002.tb00196.x 12169214 PMC6740102

[B14] Anastassiou D Genomic signal processing IEEE Signal Process Mag 2001 18 8 20

[B15] Sharma SD Shakya K Sharma SN Evaluation of DNA mapping schemes for exon detection Int Conf Comput Commun Electr Technol 2011 2011 71 74

[B16] Katoh K Misawa K Kuma K Miyata T MAFFT: a novel method for rapid multiple sequence alignment based on fast Fourier transform Nucleic Acids Res 2002 30 3059 3066 10.1093/nar/gkf436 12136088 PMC135756

[B17] Voss RF Evolution of long-range fractal correlations and 1/f noise in DNA base sequences Phys Rev Lett 1992 68 3805 3808 10.1103/PhysRevLett.68.3805 10045801

[B18] Afreixo V Bastos CAC Pinho AJ Garcia SP Ferreira PJSG Genome analysis with inter-nucleotide distances Bioinformatics 2009 25 3064 70 10.1093/bioinformatics/btp546 19759198 PMC2778338

[B19] Hota MK Srivastava VK Performance analysis of different DNA to numerical mapping techniques for identification of protein coding regions using tapered window based short-time discrete Fourier transform Int Conf Power, Control Embed Syst 2010 3 1 4

[B20] Akhtar M Epps J Ambikairajah E Signal processing in sequence analysis: advances in eukaryotic gene prediction IEEE J Sel Top Signal Process 2008 2 310 321

[B21] Saberkari H Shamsi M Sedaaghi M Golabi F Prediction of protein coding regions in DNA sequences using signal processing methods Proc. 2012 IEEE Symp. Ind. Electron. Appl. (ISIEA2012) 2012 Bandung, Indonesia 355 360

[B22] Rao KD Member S Swamy MNS Fellow L Analysis of genomics and proteomics using DSP techniques IEEE Trans Circuits Syst I Regul Pap 2008 55 370 378

[B23] Marhon SA Kremer SC Gene prediction based on DNA spectral analysis: a literature review J Comput Biol 2011 18 639 76 10.1089/cmb.2010.0184 21381961

[B24] Brigham EO Morrow RE The fast Fourier transform IEEE Spectr 1967 4 63 70

[B25] Lyons RG Understanding Digital Signal Processing 2004 Pearson Education, Upper Saddle River, NJ

[B26] Oppenheim AV Schafer RW Discrete-Time Signal Processing 2010 3 Prentice Hall, Upper Saddle River, NJ, USA

[B27] Vaidyanathan P The role of signal-processing concepts in genomics and proteomics J Franklin Inst 2004 341 111 135 10.1016/j.jfranklin.2003.12.001

[B28] Berger JA Mitra SK Carli M Neri A New approaches to genome sequence analysis based on digital signal processing IEEE Work. Genomic Signal Process. Stat. (GENSIPS) 2002 IEEE Press, Raleigh, North Carolina, USA

[B29] Tuqan J Rushdi A Member S A DSP approach for finding the codon bias in DNA sequences IEEE J Sel Top Signal Process 2008 2 343 356

[B30] Anastassiou D Frequency-domain analysis of biomolecular sequences Bioinformatics 2000 16 1073 1081 10.1093/bioinformatics/16.12.1073 11159326

[B31] Cheever EA Searls DB Karunaratne W Overton GC Using Signal Processing Techniques for DNA Sequence Comparison Proc. Fifteenth Annu. Northeast Bioeng. Conf 1989 IEEE Press, Boston, MA 173 174

[B32] Needleman SB Wunsch CD A general method applicable to the search for similarities in the amino acid sequence of two proteins J Mol Biol 1970 48 443 453 10.1016/0022-2836(70)90057-4 5420325

[B33] Robinson DF Foulds LR Comparison of phylogenetic trees Math Biosci 1981 53 131 147 10.1016/0025-5564(81)90043-2

[B34] Schliep KP phangorn: phylogenetic analysis in R Bioinformatics 2011 27 592 3 10.1093/bioinformatics/btq706 21169378 PMC3035803

[B35] Air GM Sequence relationships among the hemagglutinin genes of 12 subtypes of influenza A virus Proc Natl Acad Sci U S A 1981 78 7639 43 10.1073/pnas.78.12.7639 6174976 PMC349324

[B36] Larkin MA Blackshields G Brown NP Chenna R McGettigan PA McWilliam H Valentin F Wallace IM Wilm A Lopez R Thompson JD Gibson TJ Higgins DG Clustal W and Clustal X version 2.0 Bioinformatics 2007 23 2947 8 10.1093/bioinformatics/btm404 17846036

[B37] Sievers F Wilm A Dineen D Gibson TJ Karplus K Li W Lopez R McWilliam H Remmert M Söding J Thompson JD Higgins DG Fast, scalable generation of high-quality protein multiple sequence alignments using Clustal Omega Mol Syst Biol 2011 7 539 21988835 10.1038/msb.2011.75 PMC3261699

[B38] Kaufmann S The origins of order, vol. 209 1993 Oxford University Press, Oxford, UK 709

