# Supplementary material for: Application of discrete Fourier inter-coefficient difference for assessing genetic sequence similarity
Source: EURASIP J Bioinform Syst Biol. 2014 May 28;2014(1):8. doi: 10.1186/1687-4153-2014-8 (PMC4077688; doi:10.1186/1687-4153-2014-8)
Supplement: Additional file 1 — Application of the discrete Fourier transform on DNA for sequence similarity. Table S1. Avian Flu Sequences (FLU60). Figure S1. Histogram of % identity in FLU60. Figure S2. Alignment based dendogram for FLU60. Figure S3. FFP based dendogram for FLU60. [file 1687-4153-2014-8-S1.pdf]

Application of the Discrete Fourier Transform on DNA for Sequence Similarity

Brian R. King[1], Maurice Aburene[2], Alex Thompson[2], and Zach Warres[2]

[1]Department of Computer Science, Bucknell University, Lewisburg, PA 17837

[2] Department of Electrical Engineering, Bucknell University, Lewisburg, PA 17837

Additional File 1

This file contains large tables and figures related to the FLU60 data and related analyses.

TABLES

TABLE S1 – Avian Flu Sequences (FLU60)

The following table lists all 60 sequences used in the FLU60 dataset. This dataset contains sequences for the HA gene of the influenza virus that affected birds in the United States between January 1 and July 31, 2010.

Accession	Subtype	Details	Date Collected	Length
**CY130469**	H3N6	A/Americangreen-wingedteal/InteriorAlaska/10BM04207R0/2010	7/13/10	1740
**CY135671**	H4N6	A/Americangreen-wingedteal/InteriorAlaska/10BM05165R0/2010	7/22/10	1708
**CY135729**	H3N8	A/Americangreen-wingedteal/InteriorAlaska/10BM05376R0/2010	7/25/10	1740
**CY136043**	H4N6	A/Americangreen-wingedteal/InteriorAlaska/10BM06728R0/2010	7/30/10	1713
**CY136019**	H4N6	A/Americangreen-wingedteal/InteriorAlaska/10BM07000R0/2010	7/31/10	1708
**CY097678**	H3N8	A/Americangreen-wingedteal/Mississippi/285/2010	1/14/10	1740
**CY097686**	H11N9	A/Americangreen-wingedteal/Mississippi/300/2010	1/16/10	1709
**CY097151**	H11N9	A/Americangreen-wingedteal/Mississippi/383/2010	1/16/10	1709
**CY127913**	H6N1	A/Canadagoose/DelawareBay/34/2010	5/19/10	1709
**CY125950**	H4N6	A/mallard/California/1154/2010	7/27/10	1713
**CY094749**	H4N6	A/mallard/California/1156/2010	7/27/10	1713
**CY094773**	H4N6	A/mallard/California/1188/2010	7/29/10	1713
**CY094757**	H4N6	A/mallard/California/1210/2010	7/29/10	1713
**CY094781**	H4N6	A/mallard/California/1289/2010	7/30/10	1713

**CY120683**	H4N6	A/mallard/California/1297/2010	7/30/10	1712
**CY130364**	H10N7	A/mallard/InteriorAlaska/10BM01929R0/2010	6/15/10	1703
**CY130372**	H12N5	A/mallard/InteriorAlaska/10BM02111R0/2010	6/10/10	1712
**CY130380**	H4N6	A/mallard/InteriorAlaska/10BM02530R0/2010	6/22/10	1708
**CY130437**	H4N6	A/mallard/InteriorAlaska/10BM02644R0/2010	6/25/10	1708
**CY130453**	H9N2	A/mallard/InteriorAlaska/10BM02980R0/2010	7/2/10	1683
**CY130461**	H4N6	A/mallard/InteriorAlaska/10BM03979R0/2010	7/12/10	1708
**CY135591**	H7N3	A/mallard/InteriorAlaska/10BM04564R1/2010	7/18/10	1706
**CY135599**	H4N6	A/mallard/InteriorAlaska/10BM04618R0/2010	7/18/10	1713
**CY135623**	H4N6	A/mallard/InteriorAlaska/10BM04626R0/2010	7/18/10	1708
**CY135687**	H4N6	A/mallard/InteriorAlaska/10BM05247R0/2010	7/23/10	1708
**CY135713**	H4N6	A/mallard/InteriorAlaska/10BM05337R0/2010	7/24/10	1708
**CY135721**	H7N3	A/mallard/InteriorAlaska/10BM05347R0/2010	7/25/10	1706
**CY135811**	H3N8	A/mallard/InteriorAlaska/10BM05797R0/2010	7/26/10	1740
**CY135843**	H3N8	A/mallard/InteriorAlaska/10BM05970R0/2010	7/27/10	1740
**CY135867**	H4N6	A/mallard/InteriorAlaska/10BM06448R0/2010	7/30/10	1708
**CY135907**	H4N6	A/mallard/InteriorAlaska/10BM06456R0/2010	7/30/10	1708
**CY135947**	H3N8	A/mallard/InteriorAlaska/10BM06828R0/2010	7/31/10	1740
**CY097694**	H10N7	A/mallard/Mississippi/329/2010	1/16/10	1703
**CY097702**	H3N8	A/mallard/Mississippi/354/2010	1/16/10	1740
**CY097710**	H3N8	A/mallard/Mississippi/360/2010	1/16/10	1740
**CY097718**	H3N8	A/mallard/Mississippi/386/2010	1/17/10	1740
**CY097762**	H1N1	A/mallard/Mississippi/413/2010	1/17/10	1746
**CY097770**	H1N1	A/mallard/Mississippi/442/2010	1/19/10	1746
**CY130413**	H3N8	A/northernpintail/InteriorAlaska/10BM00303R0/2010	5/14/10	1740
**CY130477**	H3N1	A/northernpintail/InteriorAlaska/10BM00849R2/2010	5/23/10	1740
**CY130388**	H7N3	A/northernpintail/InteriorAlaska/10BM02539R0/2010	6/22/10	1706
**CY130421**	H4N6	A/northernpintail/InteriorAlaska/10BM02585R0/2010	6/22/10	1708
**CY130445**	H4N6	A/northernpintail/InteriorAlaska/10BM02791R0/2010	6/25/10	1708
**CY135639**	H4N6	A/northernpintail/InteriorAlaska/10BM04704R0/2010	7/18/10	1708
**CY135679**	H4N6	A/northernpintail/InteriorAlaska/10BM05171R0/2010	7/22/10	1708
**CY135851**	H7N3	A/northernpintail/InteriorAlaska/10BM06303R0/2010	7/29/10	1706
**CY135859**	H7N3	A/northernpintail/InteriorAlaska/10BM06306R0/2010	7/29/10	1706
**CY135939**	H7N3	A/northernpintail/InteriorAlaska/10BM06524R0/2010	7/30/10	1706
**CY136027**	H3N8	A/northernpintail/InteriorAlaska/10BM06704R0/2010	7/30/10	1740
**CY135971**	H3N8	A/northernpintail/InteriorAlaska/10BM06872R0/2010	7/31/10	1740
**CY130429**	H4N6	A/northernshoveler/InteriorAlaska/10BM02593R0/2010	6/23/10	1708
**CY130405**	H4N6	A/northernshoveler/InteriorAlaska/10BM02892R0/2010	6/30/10	1708
**CY135737**	H3N8	A/northernshoveler/InteriorAlaska/10BM05382R0/2010	7/25/10	1740
**CY135795**	H3N8	A/northernshoveler/InteriorAlaska/10BM05649R0/2010	7/26/10	1740
**CY097143**	H10N7	A/northernshoveler/Mississippi/236/2010	1/12/10	1703
**CY097670**	H10N7	A/northernshoveler/Mississippi/252/2010	1/10/10	1703
**CY097734**	H1N3	A/northernshoveler/Mississippi/397/2010	1/17/10	1746

**CY141081**	H1N1	A/red-neckedgrebe/Minnesota/AI10-1948/2010	7/7/10	1746
**CY135779**	H4N6	A/ring-neckedduck/InteriorAlaska/10BM05617R0/2010	7/26/10	1708
**CY127921**	H6N1	A/shorebird/DelawareBay/380/2010	5/20/10	1719

FIGURES

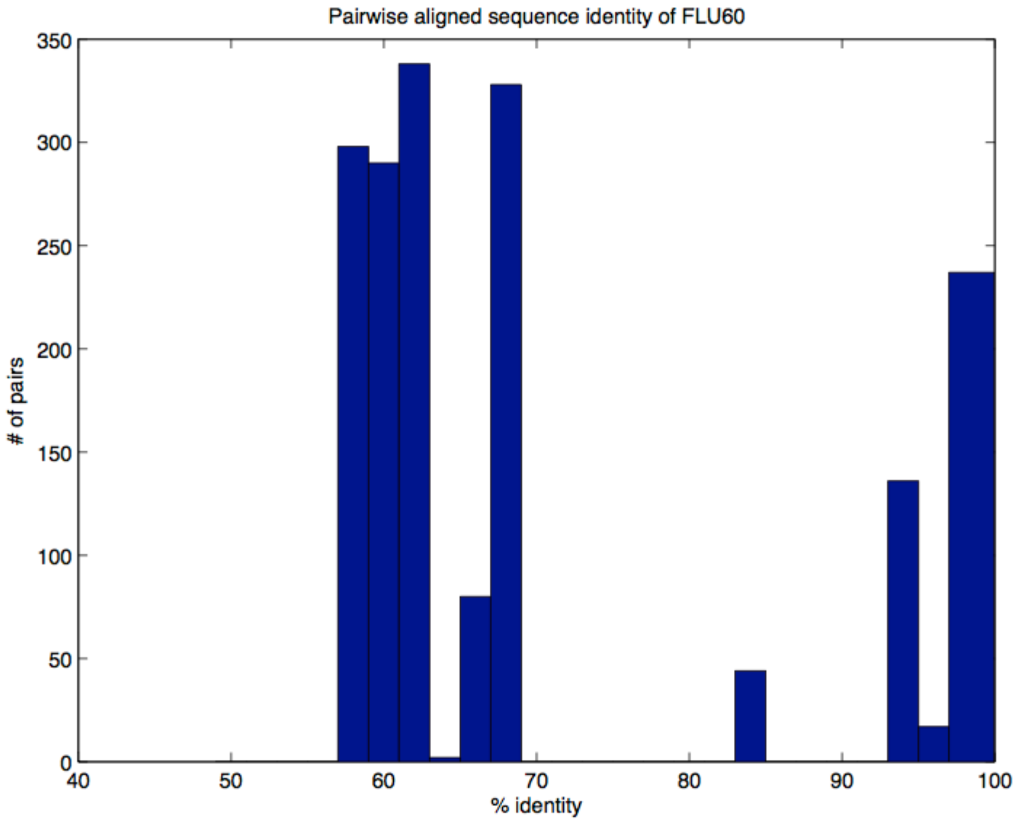

FIGURE S1 – Histogram of % identity in FLU60. The figure above shows the % sequence identity among all pairs of aligned sequences in the FLU60 dataset.

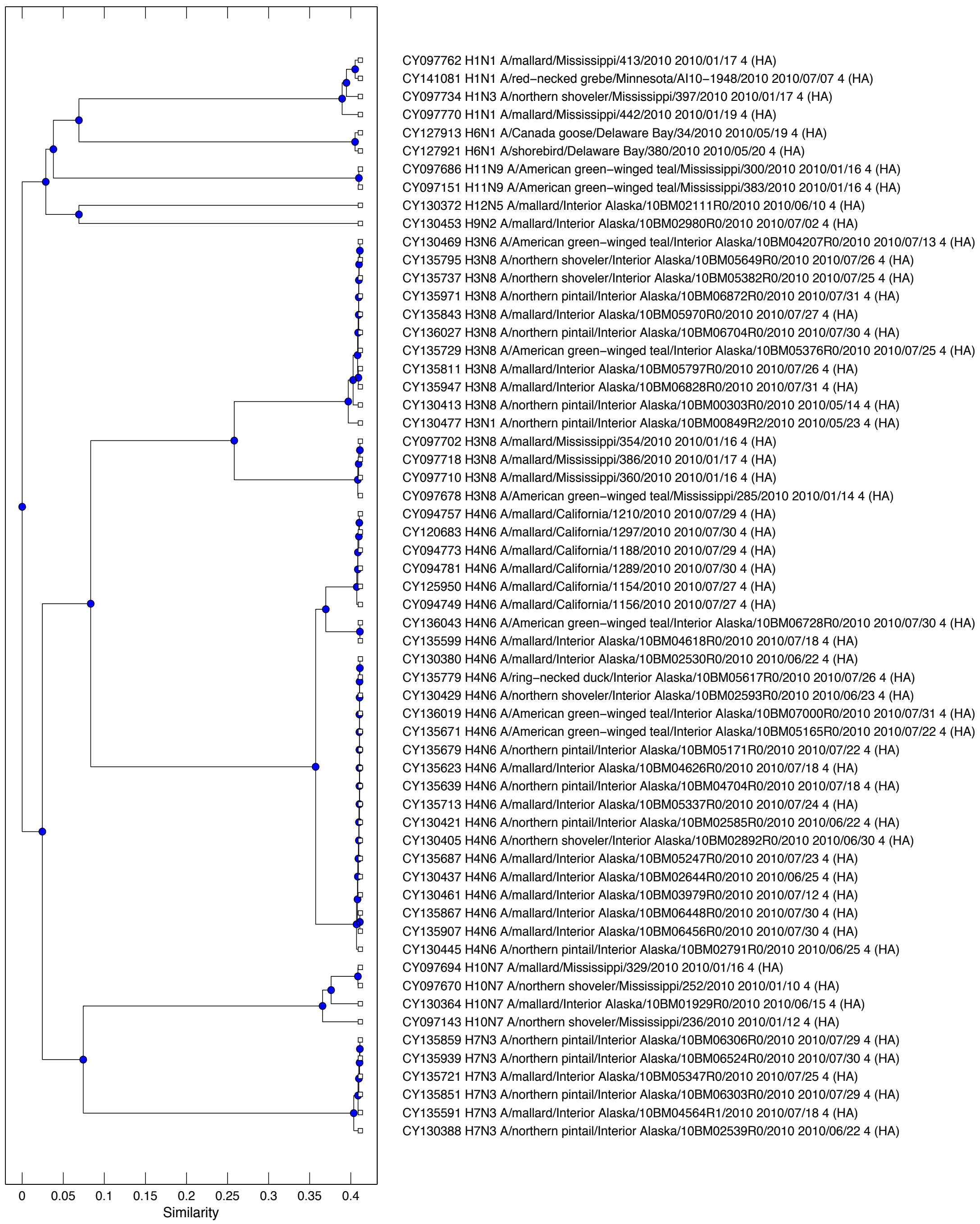

Figure S2 - Alignment based dendrogram for *FLU60*. This figure shows the resulting dendrogram generated from phylogenetic relationships inferred from pairwise alignments computed over all pairs from the *FLU60* dataset, which contains 60 HA genes from different subtypes of avian influenza, type A.

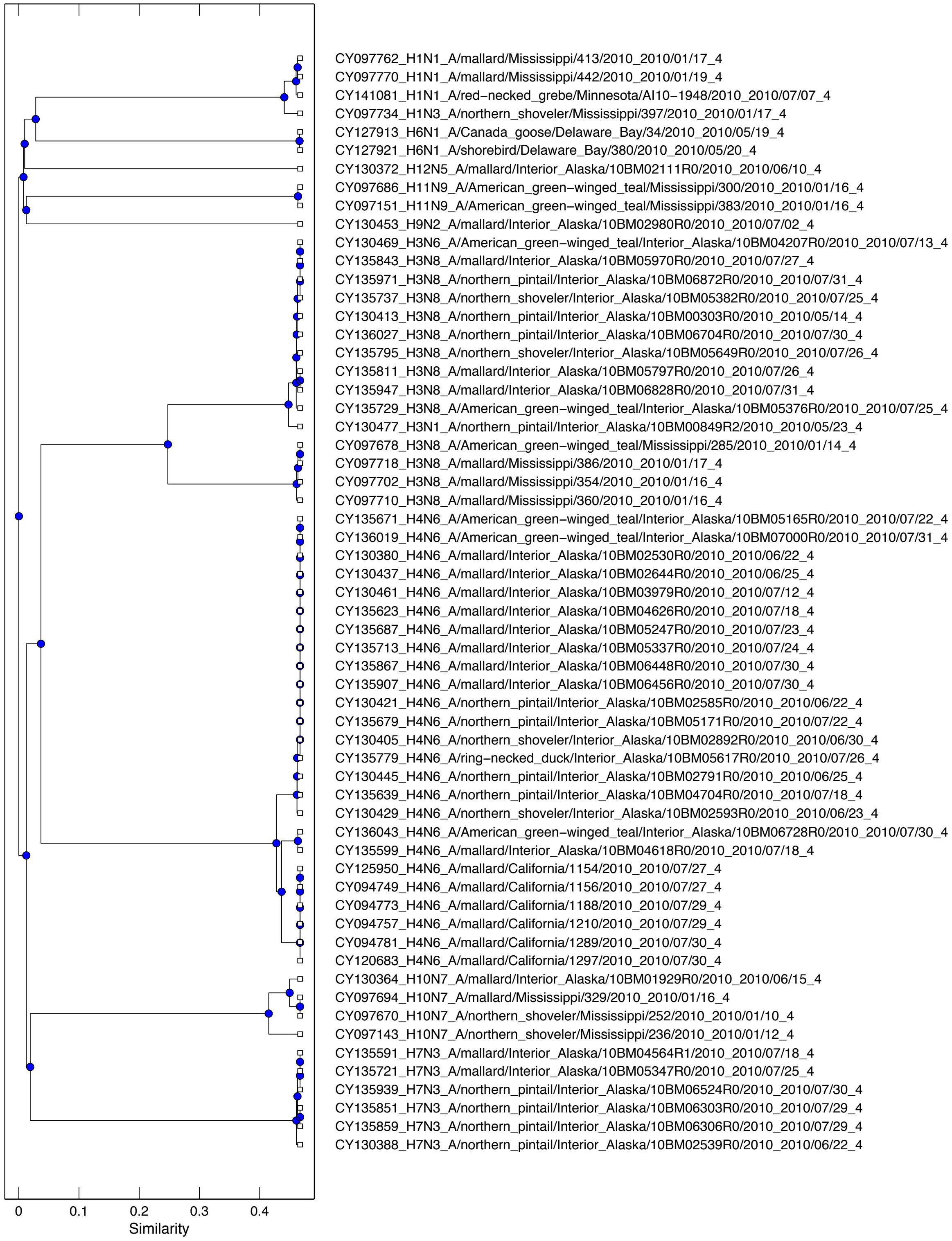

Figure S3 - FFP based dendrogram for *FLU60*. This figure shows the resulting dendrogram generated from phylogenetic relationships inferred from the FFP alignment-

free sequence method applied to the *FLU60* dataset, which contains 60 HA genes from different subtypes of avian influenza, type A.
